# Progress in Fevers

**Published:** 1900-11-17

**Authors:** 


### PROGRESS IN FEVERS.

**Malaria.**—Filkin[1] mentions the interesting fact that General Gordon was so convinced of the importance of mosquito nets as a means of keeping off malaria that he personally instructed him in the best method of making them, and insisted on a native coming with a light at night after he was in bed to kill any mosquitoes which had got under the net when he entered. He believed that the net in some way acted as a filter, whether the malarial poison was brought in by the air or by insects. In the same journal many instances are given of immunity by travellers who carefully used nets.

Koch[2] reports that many natives become immune owing to infection in childhood, and shows that numbers of infants go through attacks. He thinks that many adults, even Europeans and Chinese, attain to immunity after a time. For treatment he gives a gramme of quinine daily for two days, and then in each week for two days during two months at least. In this way relapses are prevented, but in some cases of defective absorption subcutaneous doses are necessary. Ladds at Hong Kong[3] found Anopheles in only particular pools in malarial districts where certain weeds grew, and believes a little Jeyes' Fluid is best for destroying them. Some Italian observers point out that quinine fails to act on the asexual forms, and insist that its action must be supplemented by other things, while Celli shows that immunity can be produced by methylene blue and euchinin.[4]

Veazie of New Orleans[5] warns us that the æstivo-autumnal parasite often produces cases which are mistaken for yellow fever. The face in malaria is dull; in yellow fever flushed and excited, and the eyes watery and irritable. In yellow fever there is a strawberry tongue instead of a yellow-coated one, sore throat, an enlargement of the spleen, and the temperature never falls below 99°; but jaundice and bile in the urine occur in both diseases, and in hæmaturic cases of malaria there may be albumen as in yellow fever. Black vomit may occur in either disease. In fact, an examination of the blood must be made in any doubtful case. The question of immunity raised by Koch has not been worked out in all its bearings. Manson[6] in discussing the cause of the periodicity of paroxysms thinks this depends partly on the duration of life of individual parasites and partly on the resistance of the body at certain hours of the day. When a new comer is first attacked, the disease generally takes a continued or remittent form. Later on some degree of immunity being attained, the body overcomes all parasites hatched in the stronger hours, and none survive except those starting when resistance is below par. Many animals and some few men are altogether immune, but the cause of this is unknown. The parasite, moreover, is a habitant of the deeper vessels, and comparatively few are found in the peripheral ones such as those of the finger. Hence the extraordinary anæmia caused by myriads of blood cells destroyed in the brain and other organs, while very few parasites may be seen in the blood of the finger. Hence, too, the attacks of coma, epileptiform, aphasic, hyperpyexial, or choleraic seizures which complicate malaria. Since many such extraordinary symptoms may be due simply to malaria, it is fortunate that we have three good tests for the disease: (1) If the affection shows a periodicity of 48 or 72 hours, it is malaria. Quotidian attacks may be due to syphilis, tubercle, and many other diseases. (2) Quinine properly administered for 48 or 72 hours always in malaria abates the symptoms. When, indeed, the stomach cannot absorb it, intramuscular injections of 5 or 10 grains must be given with aseptic precautions. If there is no improvement in 72 hours, the disease is not malaria. (3) The blood examined, with a $\frac{1}{12}$ objective by a trained observer, will also settle the question. Koch's dictum on quinine in hæmoglobinuria is theoretically true, but most unfortunately expressed, and has done much harm. Manson's rule is, if you find the parasites in the blood of such a case certainly give quinine; if not, do not do so; and indeed after a certain stage the parasites vanish of themselves, and there is probably no use in giving the drug then.

In other forms of malaria A. Duncan[7] at the British Medical Association meeting showed that as regards the prophylactic value of quinine the evidence was conflicting, but on the whole favourable, while careful experiments in Indian gaols supported this view, according to Buchanan. The latter has given quinine or one of its alkaloids daily to 1,600 persons for four months at a time for years without any harm. Duncan emphasises the value of rectal administration for curative purposes where the stomach fails to absorb the drug, which is confirmed by Fielding-Ould. Quinine, however, seems to have less effect on the pernicious forms of malaria than on the common ones, and the questions of dose and of the time of administration (so that it may act on the young organisms) are important. The plan adopted for treatment of the fever in the Agro Romano[8] provides for 2 grammes daily for three days, then 1 gramme for four days, then a rest for five or seven days. If no return follows half the doses are given daily for the third, fifth, and seventh weeks. The quinine is distributed among the labouring population with full instructions as a means to stamp out human sources of the disease. Since if the diseased are cured in an entire district, none of the mosquitoes in the next spring will become infected, and no new cases will appear. E. Mattei[9] slept in a most malarial region with four workmen in a mosquito-proof hut for more than a month, and reports that they all escaped the disease. The further experiments of Sambon and Lowe in dwelling among the worst districts of the Campagna with similar precautions have been widely reported. Grassi[10] treated in the same way 104 railway men and their families, rendering their houses mosquito-proof, thoroughly curing the old cases in the dead season, and giving veils and gloves to all-night watchmen. The results are said to be entirely successful. Finally, to prove that malaria could be given by infected mosquitoes to healthy persons in a non-malarial country, Dr. Manson's son allowed himself to be bitten in London by mosquitoes infected in Italy and sent by post. He developed a normal attack of fever, which was verified by examination of his blood. Fernin and Tonsini[11] exterminated all the Anopheles mosquitoes on the island of Asinara, and covered the windows of the houses with wire gauze to make doubly sure, with the result that no inhabitants had malaria. Adami[12] brings forward the question as to how it is that malaria, so common formerly in Canada, has almost disappeared except in one locality, though the drainage and physical conditions are unaltered and the Anopheles still abounds. The sources of infection and the mosquitoes were apparently unable to maintain the spread of the disease as they should according to modern theories. The same unexplained difficulty exists as to the disappearance of ague in Great Britain, where the Anopheles still occurs. However, Manson points out that only a small percentage of the mosquitoes may be infected in a given fever district, so that if the population take care to avoid being bitten often by keeping indoors at night and so on, the chance of infection may be small, and the spread of the disease may be checked. Some such unnoticed factor may exist in England and Canada. In West Africa the Liverpool Expedition found that great difficulties existed in stopping the malaria. Here, unlike what is seen in Italy, the Anopheles bite in the daytime: vast masses of natives cannot be induced to take any precautions, and travellers in the bush have to sleep in their huts or in the open. There are besides some classes of fever in which no parasites can be found, though they seem to be of malarial type. Dauber[13] gives very ably the arguments in favour of malaria being often due to a miasma from the upturned soil and infected marshes. Ross replies that no one can yet be certain that there is no other channel for the disease than the mosquito, but he thinks there is no evidence in favour of it. Chills and fatigue do produce relapses, but not infection of a specific disease. Ross and the Italians insist that mosquitoes are low fliers and seldom attack men sleeping on platforms or in upper storeys. If Dauber finds that they do so in any place, it is contrary to their usual habits. If new arrivals, though badly bitten, do not often take fever for a year or more, it is because only a few mosquitoes are infected, even of the Anopheles. Indeed, most of the bites are from Culex. A miasma would take effect at once. As to the old history of the Royal Engineers who suffered at Hong Kong while excavating, while the Highlanders escaped, the Anopheles may infest one barrack while there are none in the next; and fatigue would induce relapses in those who had suffered before. Anopheles, too, has recently been found at Hong Kong. He adds that where a crew has been attacked a week or more after leaving the coast, the fever was probably contracted on land, as there is an incubation period of something under three weeks. As to the case of England (and Canada) the fever cases have been reduced by quinine, and the Anopheles by drainage, but on the present theory both the Anopheles and the ague cases must be numerous enough for the gnat to have a fair chance of infection. At the present time the chance of an Anopheles getting a meal from an ague-stricken person and another from a healthy one is a very rare one here. The cases where an outbreak has occurred in a ship at sea during a breeze from the land, unless mosquitoes were blown on board, should be most carefully investigated; the insects may remain concealed on the ship for weeks. As to treatment, Palmer Ross, of Guiana,[14] gives a purgative and antipyrin if the temperature is over 103°, then 20 grains of quinine by mouth or rectum, then 10 grains every four hours for one day, and afterwards a daily dose at noon with arsenic three times a day. Salines are also given if fever runs high. In blackwater fever he gives 20 grains of quinine a day and arsenic. Clarac[15] thinks blackwater fever has never been seen in non-malarial persons. There is little evidence for the quinine theory, and none for that of a bacillary origin. However, secondary depressing influences precede attacks. Marchoux is in favour of the quinine theory, but confesses that it does not cause it except in persons debilitated by other causes, especially by malaria. Hence he advises quinine to prevent and cure the malaria, paradoxical as this may seem. P. Gouzien advises for its treatment injections of saline solution (3 to 10 ounces) daily till the urine clears, and in mild cases enemata of the same solution. An infusion of Cassia occidentalis is also valuable as a medicine, and it is largely grown in French territory for the purpose.

[1] Jour. Trop. Med., May. [2] Brit. Med. Jour., May. [3] Idem, p. 1,186. [4] Idem, p. 1,188. [5] New York Med. Jour., May 19. [6] Lancet, July 21. [7] Brit. Med. Jour., Sept. 1. [8] Lancet, July 7. [9] Med. Rec., Sept. 15. [10] Brit. Med. Jour., Sept. 29 [11] Idem, p. 977. [12] The Hospital, June 30. [13] Lancet, June 23. [14] Jour. Trop. Med., May, p. 257. [15] Brit. Med. Jour. Sept. 8., Ep.

